# Meningitis and endocarditis as a sequela of streptococcus pneumonia mastoiditis: A case report

**DOI:** 10.1002/ccr3.7648

**Published:** 2023-07-02

**Authors:** Mitchell Peebles, Mehnaz Roshani, Kumaraman Srivastava

**Affiliations:** ^1^ PGY‐2 Internal Medicine Resident at Texas Health Harris Methodist Hospital Fort Worth Texas USA; ^2^ Internal Medicine Core Faculty at Texas Health Harris Methodist Hospital Fort Worth Texas USA; ^3^ MS4 at Anne Burnett Marion School of Medicine at Texas Christian University Fort Worth Texas USA

**Keywords:** Austrian syndrome, endocarditis, mastoiditis, meningitis, *Streptococcus pneumonia*

## Abstract

**Key Clinical Message:**

Austrian Syndrome classically consists of meningitis, endocarditis, and pneumonia due to *Streptococcus pneumonia* bacteremia. A literature review, however, does not show variants of this triad. Our case highlights a unique variant of Austrian Syndrome with mastoiditis, meningitis, and endocarditis which requires immediate recognition and treatment to prevent devastating patient outcomes.

**Abstract:**

*Streptococcus pneumonia* is responsible for more than 50% of all bacterial meningitis and has a case fatality rate of 22% in adults. In addition, *Streptococcus pneumonia* is also one of the most common causes of acute otitis media, a known cause of mastoiditis. However, in conjunction with bacteremia and endocarditis, limited evidence is able to be identified. This sequence of infections also closely relates to Austrian syndrome. Otherwise known as Osler's triad, Austrian syndrome is a rare phenomenon of meningitis, endocarditis, and pneumonia secondary to *Streptococcus pneumonia* bacteremia that was first delineated by Robert Austrian in 1956. The incidence of Austrian syndrome is reported to be less than <0.0001% per year and has decreased significantly since the initial usage of penicillin in 1941. Despite this, the mortality rate of Austrian syndrome is still around 32%. Despite an extensive literature review, we were unable to find any reported cases of variants of Austrian syndrome that include mastoiditis as the primary insult. As such, we present a unique presentation of Austrian syndrome with mastoiditis, endocarditis, and meningitis with complex medical management that led to resolution for the patient. To discuss the presentation, progression, and complex medical management of a previously undiscussed triad of mastoiditis, meningitis, and endocarditis occurring in a patient.

## 1 CASE REPORT

**FIGURE 1:**
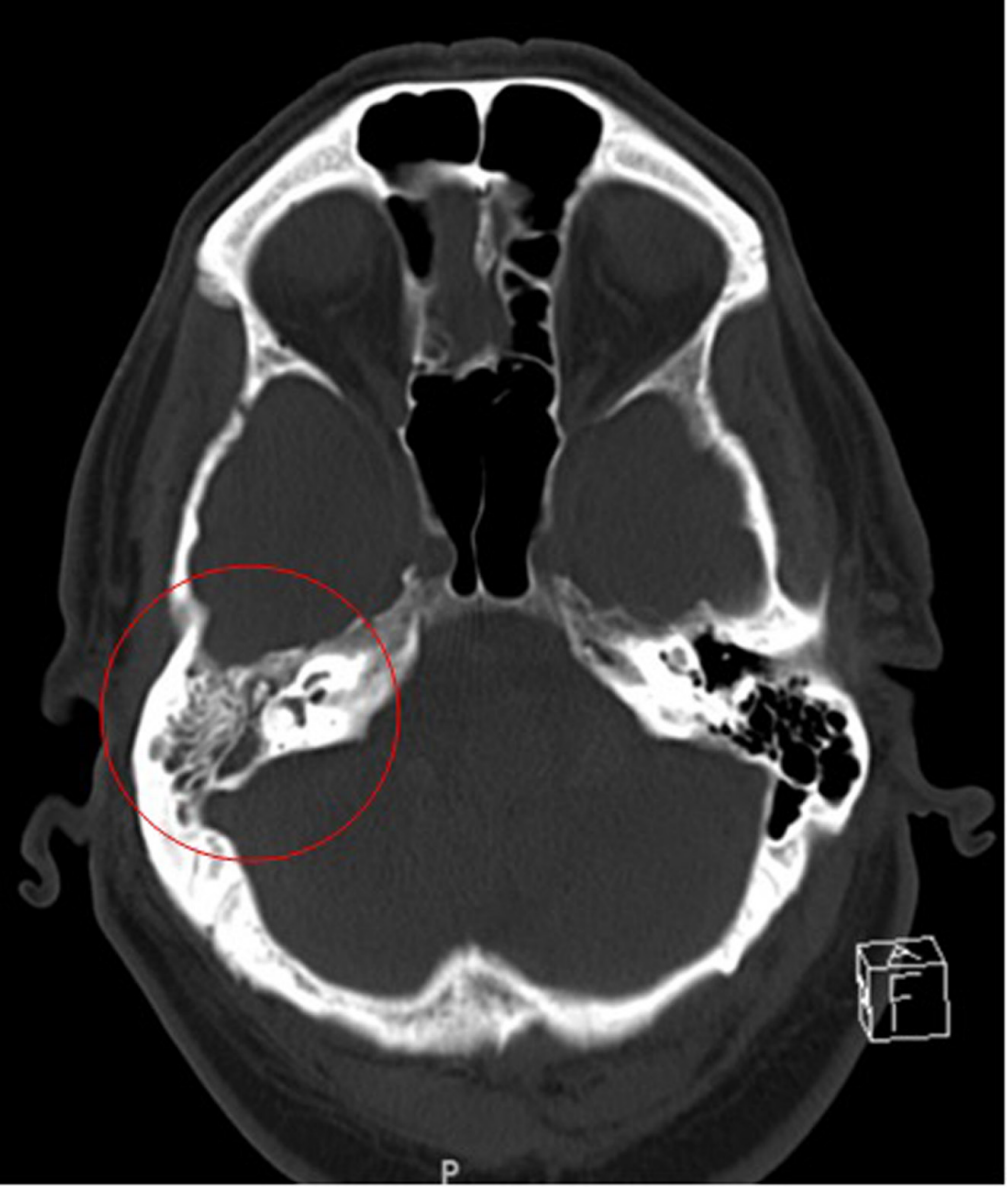
CT head without contrast revealing a right sided tympanomastoid effusion.

**FIGURE 2:**
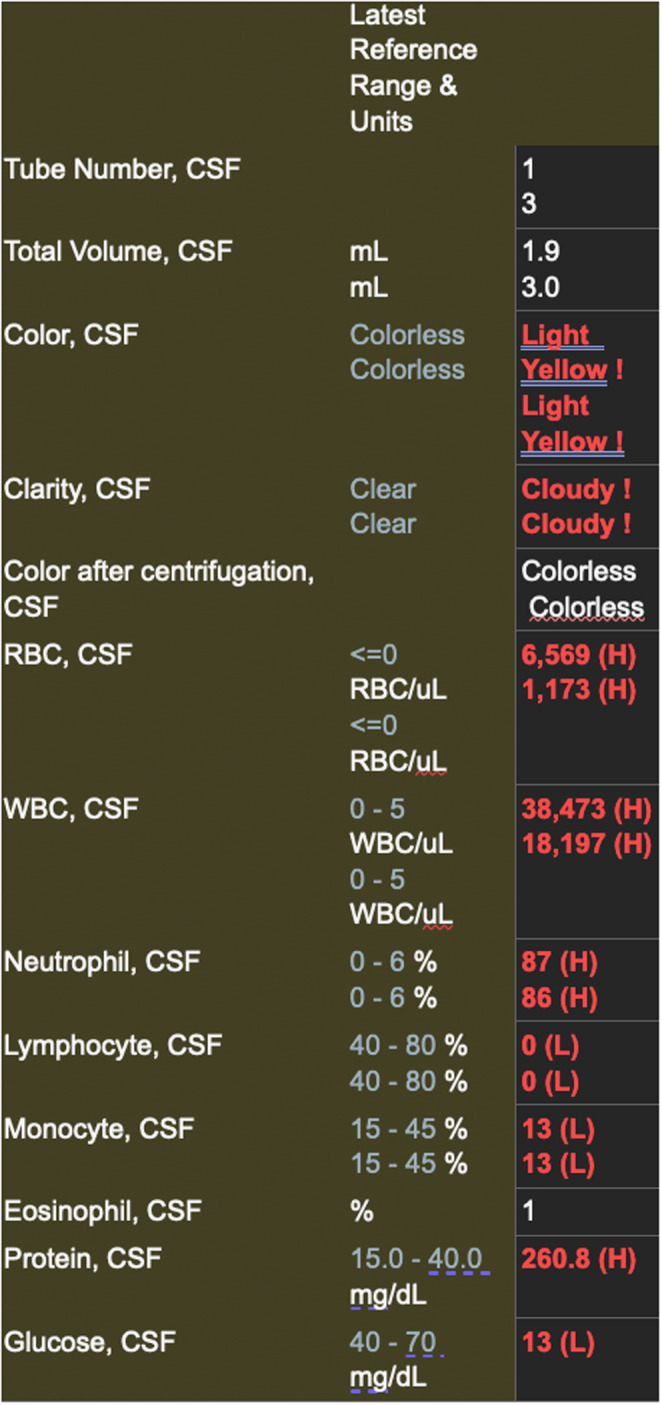
Results of the lumbar puncture consistent with bacterial meningitis.

A 78‐year‐old man with a past medical history of resected prostate cancer, pheochromocytoma, and obstructive sleep apnea presented to the emergency department due to altered mental status, right‐sided weakness, and expressive aphasia. The patient's wife reported that the patient was shivering and lethargic. His last known normal was determined to be 14 h prior to presentation. The patient's recent medical history was remarkable for a planned procedure to undergo bilateral eustachian tube placement due to a recent history of frequent ear infections. The patient developed a fever of 103 F (39.4 C) in the emergency department and initial labs revealed a leukocytosis of 12 K/uL with a lactic acid of 1.67 mmol/L. Further imaging included CT scan of the head as well as CT angiography of the head and neck which did not reveal any acute abnormalities other than a right‐sided tympanomastoid effusion (Figure 1). The physical exam on admission was significant for altered mental status, agitation, and subjective nuchal rigidity. Moreover, the patient was incoherent, and had a significant decline from his baseline independence according to his wife. The patient received a fluid bolus of 2600 mL (30 mL/kg of body weight) and 2 g of cefepime in the ED prior to hospital admission. An attempt to obtain a lumbar puncture in the ED was unsuccessful due to the patient's increased agitation. Upon admittance to the hospital, 2 g of vancomycin and ceftriaxone were administered to the patient for empiric coverage of meningitis. Blood cultures were positive for *Streptococcus pneumonia*. On hospital day 2, another attempt at lumbar puncture under fluoroscopy was attempted but was unsuccessful due to ongoing patient agitation. Lumbar puncture was finally obtained on hospital day 3 under sedation with the following cerebrospinal fluid findings consistent with bacterial meningitis (Figure 2).

CSF cultures showed no growth and otolaryngology was consulted for further evaluation of right tympanomastoid effusion secondary to mastoiditis. The patient underwent right mastoidectomy with bilateral myringotomy and bilateral ear tube insertion on hospital day 3. On pathological examination, the mastoid bone was noted to be sclerotic, and the mastoid air cells were filled with inflammatory tissue. Vancomycin was also discontinued on hospital day 4 after cultures revealed sensitivity to ceftriaxone.

**FIGURE 3:**
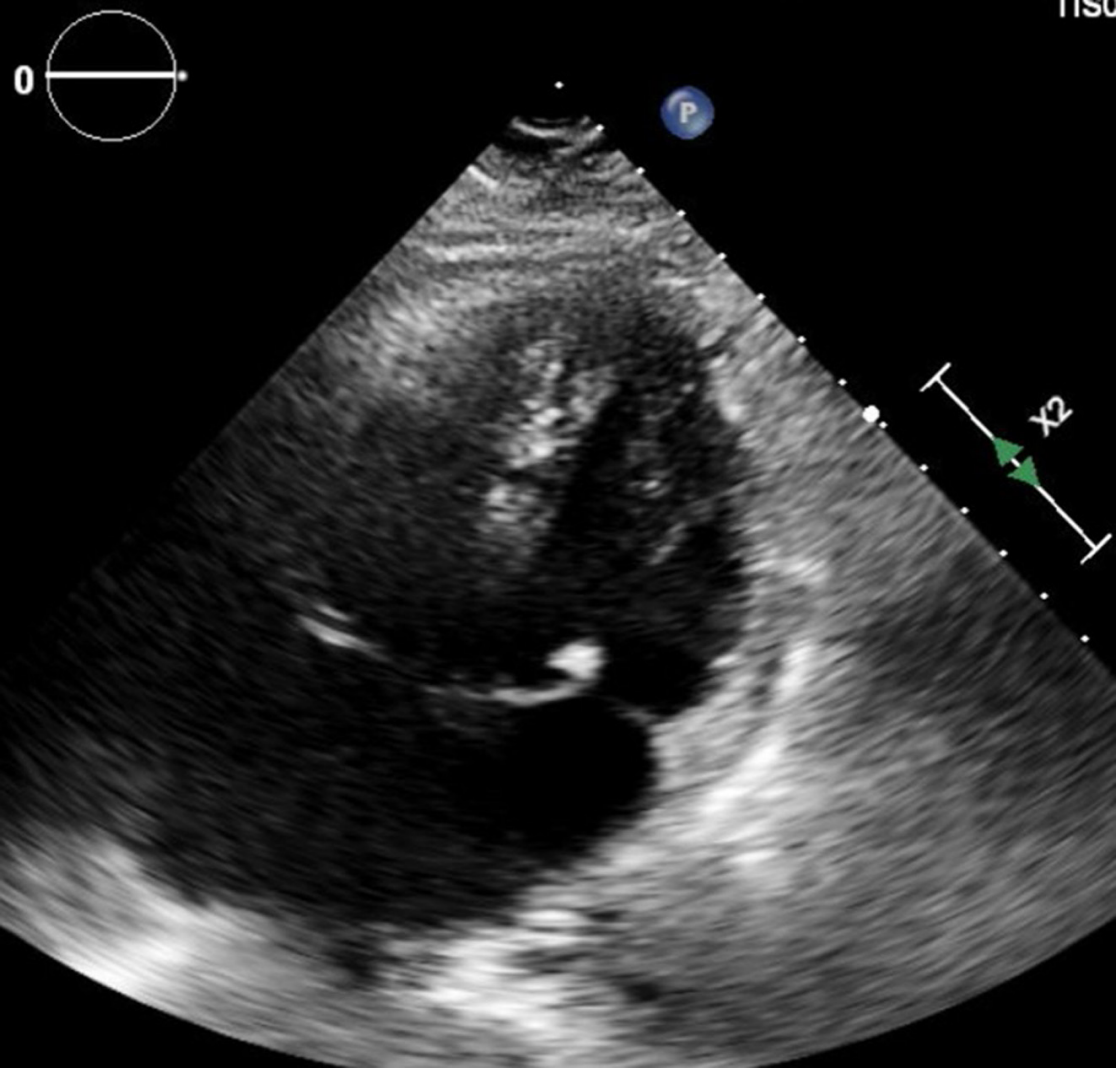
Complete transthoracic echocardiogram—apical four chamber view showing a Mitral valve with round immobile echodensity associated with chordal structures of anterior mitral leaflet.

Further workup included a transthoracic echocardiogram which revealed an immobile mitral valve echo‐density measuring 0.7 cm in diameter associated with chordal structures of the anterior mitral leaflet without evidence of mitral regurgitation or mitral stenosis (Figure 3). This imaging strongly suggested endocarditis. Infectious diseases and cardiology were consulted and determined transesophageal echocardiogram was not required to confirm the diagnosis. The decision was made to extend the patient's ceftriaxone for 6 weeks for treatment of endocarditis. However, the patient developed significant surgical site bleeding on hospital day 6 with an elevated prothrombin time of 16.2 sec and platelet count of 111 K/uL which was initially presumed to be due to ceftriaxone‐induced thrombocytopenia. Therefore, ceftriaxone was stopped, and vancomycin was restarted. The patient's prothrombin time continued to be elevated at 18 sec with a platelet count of 56 K/uL on hospital day 7 and 17.3 sec with a platelet count of 71 K/uL on hospital day 8. Furthermore, the patient also had depleted fibrinogen levels at 62 mg/dL on hospital day 7 and 145 mg/dL on hospital day 8 suggesting that postoperative disseminated intravascular coagulation was the cause of the patient's bleeding rather than ceftriaxone‐induced thrombocytopenia. Thus, two units of cryoprecipitate were given, and the patient's fibrinogen levels stabilized at 156 mg/dL with a platelet count of 216 K/uL after hospital day 9. The patient had deconditioned significantly from his baseline status so physical therapy was initiated and the patient responded very well. On hospital day 10, a peripherally inserted central catheter line was placed so that the patient could continue to receive intravenous vancomycin 1.5 g every 12 h at home. The patient was stable for home discharge on hospital day 11.

## 2 DISCUSSION/CONCLUSION

Austrian syndrome has been described as a triad of meningitis, endocarditis, and pneumonia with multiple case reports and case series describing the difficulties of managing this complex disease.^1^, ^2^, ^3^ There are, however, no published cases that demonstrate a variant of Austrian syndrome with the triad of mastoiditis, endocarditis, and meningitis. Our patient initially presented with altered mental status with multiple failed attempts at a lumbar puncture due to agitation. So, we initiated empiric coverage of meningitis with vancomycin and ceftriaxone as broad spectrum antibiotics should be initiated for suspected meningitis even if confirmatory CSF results are not available.^4^ Notably, we did not add ampicillin because our clinical suspicion of *Listeria monocytogenes* was low. Our patient's right tympanomastoid effusion was assumed to be the nidus for the initial infection and, thus, we consulted ENT since the patient did not improve clinically despite receiving appropriate antibiotics.^5^ After the surgery was completed, our patient began to show signs of improvement suggesting that source control is critical to managing a complex disease process like Austrian syndrome. Moreover, our patient's meningitis likely occurred as a sequela of his mastoiditis which has previously been described in several cases as a rather uncommon complication of mastoiditis more typically associated with children but also reported in adults albeit infrequently.^6^, ^7^ As further demonstrated by this literature, otogenic meningitis does not respond to conventional antibiotics, and requires surgical removal of infected tissue prior to complete resolution.

Furthermore, the etiology of our patient's endocarditis may have been due to meningitis as research has shown that there is a 2% incidence rate of endocarditis with meningitis.^8^ A thorough literature review revealed only one other case report that demonstrated that mastoiditis may also directly cause endocarditis.^9^ Our patient further underwent a transthoracic echocardiogram because of the presence of *Streptococcus pneumonia* bacteremia. If the TTE had returned negative, we would have proceeded with a transesophageal echocardiogram since it is more sensitive for endocarditis and our clinical suspicion for endocarditis was high.^10^ Therefore, it is critical to recognize the possible presence of Austrian syndrome even if all three elements of the classic triad are not present as early recognition significantly improves patient outcomes.^11^ Unfortunately, our patient also developed postoperative DIC which is a well‐known complication of both meningitis and head/neck surgery^12^, ^13^ and was successfully treated with two units of cryoprecipitate.

Our patient endured several complications due to his complex interaction of diseases, but we were able to treat him effectively. As such, this case report presents an exceptionally distinct variant of a rare phenomenon in Austrian syndrome with complex medical management that resulted in complete recovery of the patient.

## AUTHOR CONTRIBUTIONS

**Mitchell Peebles:** Formal analysis; investigation; writing – original draft; writing – review and editing. **Mehnaz Roshani:** Supervision; writing – review and editing. **Kumaraman Srivastava:** Formal analysis; investigation; methodology; writing – original draft; writing – review and editing.

## CONFLICT OF INTEREST STATEMENT

Authors state no conflicts of interest.

## ETHICS STATEMENT

The authors of this study had the required consent from the patient involved in this case report.

## Data Availability

Data sharing is not applicable to this article as no new data were created or analyzed in this study.
